# Supplementary material for: Perinatal Outcomes at Birth in Women Infected and Non-Infected with SARS-CoV-2: A Retrospective Study
Source: Healthcare (Basel). 2023 Oct 27;11(21):2833. doi: 10.3390/healthcare11212833 (PMC10648606; doi:10.3390/healthcare11212833)
Supplement: Supplementary file 1 [file healthcare-11-02833-s001.zip › healthcare-2616319-supplementary materials.pdf]

Supplementary Table S1. Comparison of the characteristics between the unvaccinated and vaccinated women (N = 2676).

		Unvaccinated		Vaccinated			
		Mean/n	DS/%	Mean/n	DS/%	OR (95%CI)	*p*-value
Maternal age		30.3	6.3	32.1	6.5	-	0.272
Country of origin	Spain	1477	69.5	445	80.9	0.5 (0.4-.07)	<0.001
	Foreign	649	30.5	105	19.1		
Parity	Primiparous	1154	54.3	330	60.0	0.8 (0.7-0.9)	0.016
	Multiparous	972	45.7	220	40.0		
Preterm birth <37 weeks	No	1971	92.7	495	90.0	1.4 (1.0-1.9)	0.035
	Yes	155	7.3	55	10.0		
Premature preterm rupture of membranes	No	1263	59.4	301	54.7	1.2 (1.0-1.5)	0.047
	Yes	863	40.6	249	45.3		
Placental abruption	No	2125	100.0	549	99.8	3.8 (0.2-61.9)	0.303
	Yes	1	0.0	1	0.2		
Antepartum hemorrhage	No	2109	99.2	542	98.5	1.8 (0.8-4.3)	0.155
	Yes	17	0.8	8	1.5		
Postpartum hemorrhage	No	2116	99.5	539	98.0	4.3 (1.8-10.2)	<0.001
	Yes	10	0.5	11	2.0		
Caesarean section	No	1711	80.5	438	79.6	1.1 (0.8-1.3)	0.658
	Yes	415	19.5	112	20.4		
Operative delivery	No	1872	88.1	486	88.4	0-9 (0.7-1.3)	0.841
	Yes	254	11.9	64	11.6		
Fetal distress (defined by healthcare provider)	No	2097	98.6	545	99.1	0.6 (0.3-1.7)	0.396
	Yes	29	1.4	5	0.9		
Fetal growth restriction	No	2090	98.3	540	98.2	1.1 (0.5-2.2)	0.841
	Yes	36	1.7	10	1.8		
Preeclampsia/eclampsia/gestational hypertension/HELLP	No	2073	97.5	522	94.9	2.1 (1.3-3.3)	0.02
	Yes	53	2.5	28	5.1		
Gestational diabetes	No	1973	92.8	506	92.0	1.1 (0.8-1.6)	0.52
	Yes	153	7.2	44	8.0		
Chorioamnionitis/endometritis	No	2116	99.5	545	99.1	1.9 (0.6-5.7)	0.219
	Yes	10	0.5	5	0.9		
Maternal sepsis	No	2126	100.0	550	100.0	-	NA
	Yes	0	0.0	0	0.0		
Maternal Blood transfusion	No	2124	99.9	550	100.0	-	NA
	Yes	2	0.1	0	0.0		
Maternal death	No	2126	100.0	550	100.0	-	NA
	Yes	0	0.0	0	0.0		

Presenting signs/symptoms (if available)	No	2125	100.0	547	99.5	11.7 (1.2-112.6)	0.007
	Yes	1	0.0	3	0.5		
Intensive care unit admission	No	2116	99.5	545	99.1	1.9 (0.7-5.7)	0.219
	Yes	10	0.5	5	0.9		
Days in ICU	0	2117	99.6	545	99.1	-	NA
	1	6	0.3	4	0.7		
	2	3	0.1	0	0.0		
	4	0	0.0	1	0.2		
Intubation	No	2126	100.0	550	100.0	-	NA
	Yes	0	0.0	0	0.0		
Supplemental oxygen	No	2123	99.9	546	99.3	5.2 (1.2-23.2)	0.016
	Yes	3	0.1	4	0.7		
Cardiac manifestation: Myocardial infarction, cardiomyopathy, arrhythmia	No	2126	100.0	550	100.0	-	NA
	Yes	0	0.0	0	0.0		
Neurologic manifestation: seizures, hemorrhagic or ischemic stroke, coma, other	No	2126	100.0	550	100.0	-	NA
	Yes	0	0.0	0	0.0		
Thrombotic manifestation: deep vein thrombosis, pulmonary embolism, arterial thrombosis, other	No	2126	100.0	550	100.0	-	NA
	Yes	0	0.0	0	0.0		
Coagulopathy	No	2126	100.0	550	100.0	-	NA
	Yes	0	0.0	0	0.0		
Small for gestational age	No	2064	97.1	536	97.5	0.9 (0.5-1.6)	0.641
	Yes	62	2.9	14	2.5		
Large for gestational age	No	1862	87.6	492	89.5	0.8 (0.6-1.1)	0.229
	Yes	264	12.4	58	10.5		
Apgar < 7 at 5 minutes	No	2110	99.2	547	99.5	0.7 (0.2-2.5)	0.606
	Yes	16	0.8	3	0.5		
NICU admission	No	1958	92.1	507	92.2	0.9 (0.7-1.4)	0.948
	Yes	168	7.9	43	7.8		
Length of stay > 4 days	No	2103	98.9	544	98.9	1.0 (0.4-2.5)	0.985
	Yes	23	1.1	6	1.1		
Respiratory distress	No	2094	98.5	543	98.7	0.8 (0.4-1.9)	0.685
	Yes	32	1.5	7	1.3		
Ventilator support	No	2097	98.6	542	98.5	1.0 (0.5-2.3)	0.871
	Yes	29	1.4	8	1.5		
Neonatal anemia	No	2125	100.0	550	100.0	-	NA
	Yes	1	0.0	0	0.0		
Neonatal Sepsis	No	2123	99.9	550	100.0	-	NA

	Yes	3	0.1	0	0.0		
Neonatal SARS-CoV-2 infection	No	2126	100.0	550	100.0	-	NA
	Yes	0	0.0	0	0.0		
Congenital anomaly	No	2121	99.8	548	99.6	1.5 (0.3-8.0)	0.599
	Yes	5	0.2	2	0.4		
Hypoxic ischemic encephalopathy	No	2121	99.8	549	99.8	0.8 (0.1-6.6)	0.814
	Yes	5	0.2	1	0.2		
Neonatal death	No	2123	99.9	547	99.5	3.8 (0.8-19.2)	0.074
	Yes	3	0.1	3	0.5		

*Chi-squared test; ‡ Student T test

ICU: intensive care unit; NICU: neonatal intensive care unit; HELLP: hemolysis, elevated liver enzymes, and a low platelet count. NA: not apply.

Supplementary Table S2. Comparison of the characteristics between the unvaccinated and vaccinated women infected to SARS-CoV-2 (*n* = 52).

		Infected				
		Unvaccinated (*n* = 26)		Vaccinated (*n* = 26)		
		Mean/n	DS/%	Mean/n	DS/%	*p*-value*
Maternal age		32	6.8	30	6.3	0.271
Parity	Primiparous	8	30.8	13	50.0	0.158
	Multiparous	18	69.2	13	50.0	
Country of origin	Spain	17	65.4	19	73.1	0.548
	Foreign	9	34.6	7	26.9	
Preterm birth < 37 weeks	No	24	92.3	23	88.5	0.638
	Yes	2	7.7	3	11.5	
Premature preterm rupture of membranes	No	22	84.6	19	73.1	0.308
	Yes	4	15.4	7	26.9	
Antepartum hemorrhage	No	26	100.0	25	96.2	NA
	Yes	0	0.0	1	3.8	
Postpartum hemorrhage	No	26	100.0	25	96.2	NA
	Yes	0	0.0	1	3.8	
Caesarean section	No	24	92.3	20	76.9	0.124
	Yes	2	7.7	6	23.1	
Operative delivery	No	22	84.6	20	76.9	0.482
	Yes	4	15.4	6	23.1	
Fetal distress (defined by healthcare provider)	No	24	92.3	26	100.0	NA
	Yes	2	7.7	0	0.0	
Preeclampsia/eclampsia/gestational hypertension/HELLP	No	26	100.0	25	96.2	0.313
	Yes	0	0.0	1	3.8	
Gestational diabetes	No	26	100.0	22	84.6	NA
	Yes	0	0.0	4	15.4	
Chorioamnionitis/endometritis	No	25	96.2	26	100.0	NA
	Yes	1	3.8	0	0.0	
Presenting signs/symptoms (if available)	No	25	96.2	23	88.5	0.298
	Yes	1	3.8	3	11.5	
Intensive care unit admission	No	26	100.0	25	96.2	NA
	Yes	0	0.0	1	3.8	
Days in ICU	0	26	100.0	25	96.2	NA
	1	0	0.0	0	0.0	

	2	0	0.0	0	0.0	
	4	0	0.0	1	3.8	
Supplemental oxygen	No	26	100.0	25	96.2	NA
	Yes	0	0.0	1	3.8	
Small for gestational age	No	26	100.0	24	92.3	NA
	Yes	0	0.0	2	7.7	
Large for gestational age	No	25	96.2	23	88.5	0.298
	Yes	1	3.8	3	11.5	
NICU admission	No	23	88.5	20	76.9	0.271
	Yes	3	11.5	6	23.1	
Length of stay > 4 days	No	25	96.2	24	92.3	0.552
	Yes	1	3.8	2	7.7	
Respiratory distress	No	25	96.2	25	96.2	1
	Yes	1	3.8	1	3.8	
Ventilator support	No	25	96.2	25	96.2	1
	Yes	1	3.8	1	3.8	
Congenital anomaly	No	26	100.0	24	92.3	NA
	Yes	0	0.0	2	7.7	

*Chi-squared test; ICU: intensive care unit; NICU: neonatal intensive care unit; HELLP: hemolysis, elevated liver enzymes, and a low platelet count; NA: not apply.

Supplementary Table S3. Descriptive statistics by vaccination status (unvaccinated, incomplete regimen, complete regimen). Odds ratios were calculated by means of a multinomial model.

		Vaccination status			Incomplete vs. Unvaccinated		Complete vs. Unvaccinated	
	Total N = 2676	Unvaccinated *n* = 2126	Incomplete regimen *n* = 108	Complete regimen *n* = 442	OR (95% CI)[1]	*p*-value	OR (95% CI)[1]	*p*-value
Maternal age					1.0 (0.9; 1.0)	0.035	1.0 (1.0; 1.1)	<0.001
Mean (SD)	31.19 (6.12)	31.02 (6.16)	29.74 (5.89)	32.38 (5.84)				
Median (IQR)	32.00 (27.00, 36.00)	32.00 (27.00, 35.00)	30.00 (25.00, 35.00)	33.00 (29.00, 37.00)				
Range	14.00, 50.00	15.00, 50.00	18.00, 44.00	14.00, 47.00				
Parity								
Primiparous	1 484 (55%)	1 154 (54%)	51 (47%)	279 (63%)	1 (ref.)		1 (ref.)	
Multiparous	1 192 (45%)	972 (46%)	57 (53%)	163 (37%)	1.3 (0.9; ---)	0.152	0.7 (0.6; 0.9)	<0.001
Country of origin								
Spain	1 922 (72%)	1 477 (69%)	99 (92%)	346 (78%)	1 (ref.)		1 (ref.)	
Foreign	754 (28%)	649 (31%)	9 (8.3%)	96 (22%)	0.2 (0.1; 0.4)	<0.001	0.6 (0.5; 0.8)	<0.001
Preterm birth <37 weeks								
No	2 466 (92%)	1 971 (93%)	96 (89%)	399 (90%)	1 (ref.)		1 (ref.)	
Yes	210 (7.8%)	155 (7.3%)	12 (11%)	43 (9.7%)	1.6 (0.9; ---)	0.144	1.4 (1.0; ---)	0.082
Premature rupture of membranes								

		Vaccination status			Incomplete vs. Unvaccinated		Complete vs. Unvaccinated	
	Total<br>N = 2676	Unvaccinated<br>*n* = 2126	Incomplete regimen<br>*n* = 108	Complete regimen<br>*n* = 442	OR (95% CI)[1]	*p*-value	OR (95% CI)[1]	*p*-value
No	1 564 (58%)	1 263 (59%)	53 (49%)	248 (56%)	1 (ref.)		1 (ref.)	
Yes	1 112 (42%)	863 (41%)	55 (51%)	194 (44%)	1.5 (1.0; ---)	0.034	1.1 (0.9; 1.4)	0.200
Antepartum hemorrhage								
No	2 651 (99%)	2 109 (99%)	107 (99%)	435 (98%)	1 (ref.)		1 (ref.)	
Yes	25 (0.9%)	17 (0.8%)	1 (0.9%)	7 (1.6%)	1.2 (0.2; ---)	0.886	2.0 (0.8; ---)	0.126
Postpartum hemorrhage								
No	2 655 (99%)	2 116 (100%)	107 (99%)	432 (98%)	1 (ref.)		1 (ref.)	
Yes	21 (0.8%)	10 (0.5%)	1 (0.9%)	10 (2.3%)	2.0 (0.3; ---)	0.517	4.9 (2.0; ---)	<0.001
Caesarean section								
No	2 149 (80%)	1 711 (80%)	90 (83%)	348 (79%)	1 (ref.)		1 (ref.)	
Yes	527 (20%)	415 (20%)	18 (17%)	94 (21%)	0.8 (0.5; 1.4)	0.465	1.1 (0.9; 1.4)	0.402
Instrumental delivery								
No	2 358 (88%)	1 872 (88%)	95 (88%)	391 (88%)	1 (ref.)		1 (ref.)	
Yes	318 (12%)	254 (12%)	13 (12%)	51 (12%)	1.0 (0.6; 1.8)	0.978	1.0 (0.7; 1.3)	0.809
Fetal distress								
No	2 642 (99%)	2 097 (99%)	108 (100%)	437 (99%)	1 (ref.)		1 (ref.)	

		Vaccination status			Incomplete vs. Unvaccinated		Complete vs. Unvaccinated	
	Total<br>N = 2676	Unvaccinated<br>*n* = 2126	Incomplete regimen<br>*n* = 108	Complete regimen<br>*n* = 442	OR (95% CI)[1]	*p*-value	OR (95% CI)[1]	*p*-value
Yes	34 (1.3%)	29 (1.4%)	0 (0%)	5 (1.1%)	0.0 (0.0; 1 023 228 218 248)	0.723	0.8 (0.3; ---)	0.697
**Preeclampsia / eclampsia / gestational hypertension / HELLP**								
No	2 595 (97%)	2 073 (98%)	102 (94%)	420 (95%)	1 (ref.)		1 (ref.)	
Yes	81 (3.0%)	53 (2.5%)	6 (5.6%)	22 (5.0%)	2.3 (1.0; ---)	0.060	2.0 (1.2; ---)	0.006
**Gestational diabetes**								
No	2 479 (93%)	1 973 (93%)	100 (93%)	406 (92%)	1 (ref.)		1 (ref.)	
Yes	197 (7.4%)	153 (7.2%)	8 (7.4%)	36 (8.1%)	1.0 (0.5; ---)	0.934	1.1 (0.8; 1.7)	0.488
**Chorioamnionitis/endometritis**								
No	2 661 (99%)	2 116 (100%)	107 (99%)	438 (99%)	1 (ref.)		1 (ref.)	
Yes	15 (0.6%)	10 (0.5%)	1 (0.9%)	4 (0.9%)	2.0 (0.3; ---)	0.517	1.9 (0.6; ---)	0.267
**Presence of SARS-CoV-2 signs/symptoms**								
No	2 672 (100%)	2 125 (100%)	107 (99%)	440 (100%)	1 (ref.)		1 (ref.)	
Yes	4 (0.1%)	1 (<0.1%)	1 (0.9%)	2 (0.5%)	20 (1.2; ---)	0.035	9.7 (0.9; ---)	0.064
**ICU admission**								

		Vaccination status			Incomplete vs. Unvaccinated		Complete vs. Unvaccinated	
	Total<br>N = 2676	Unvaccinated<br>*n* = 2126	Incomplete regimen<br>*n* = 108	Complete regimen<br>*n* = 442	OR (95% CI)[1]	*p*-value	OR (95% CI)[1]	*p*-value
No	2 661 (99%)	2 116 (100%)	106 (98%)	439 (99%)	1 (ref.)		1 (ref.)	
Yes	15 (0.6%)	10 (0.5%)	2 (1.9%)	3 (0.7%)	4.0 (0.9; ---)	0.077	1.4 (0.4; ---)	0.577
Days in ICU					3.7 (0.6; ---)	0.154	0.5 (0.0; ---)	0.620
Mean (SD)	1.33 (0.90)	1.20 (0.63)	2.50 (2.12)	1.00 (0.00)				
Median (IQR)	1.00 (1.00, 1.50)	1.00 (1.00, 1.75)	2.50 (1.75, 3.25)	1.00 (1.00, 1.00)				
Range	0.00, 4.00	0.00, 2.00	1.00, 4.00	1.00, 1.00				
Missing	2 661	2 116	106	439				
Supplemental oxygen								
No	2 669 (100%)	2 123 (100%)	106 (98%)	440 (100%)	1 (ref.)		1 (ref.)	
Yes	7 (0.3%)	3 (0.1%)	2 (1.9%)	2 (0.5%)	13 (2.2; ---)	0.005	3.2 (0.5; ---)	0.201
Small for gestational age								
No	2 600 (97%)	2 064 (97%)	106 (98%)	430 (97%)	1 (ref.)		1 (ref.)	
Yes	76 (2.8%)	62 (2.9%)	2 (1.9%)	12 (2.7%)	0.6 (0.2; ---)	0.521	0.9 (0.5; 1.7)	0.818
Large for gestational age								
No	2 354 (88%)	1 862 (88%)	94 (87%)	398 (90%)	1 (ref.)		1 (ref.)	
Yes	322 (12%)	264 (12%)	14 (13%)	44 (10.0%)	1.1 (0.6; 1.9)	0.867	0.8 (0.6; 1.1)	0.148

		Vaccination status			Incomplete vs. Unvaccinated		Complete vs. Unvaccinated	
	Total<br>N = 2676	Unvaccinated<br>*n* = 2126	Incomplete regimen<br>*n* = 108	Complete regimen<br>*n* = 442	OR (95% CI)[1]	*p*-value	OR (95% CI)[1]	*p*-value
NICU admission								
No	2 465 (92%)	1 958 (92%)	101 (94%)	406 (92%)	1 (ref.)		1 (ref.)	
Yes	211 (7.9%)	168 (7.9%)	7 (6.5%)	36 (8.1%)	0.8 (0.4; 1.8)	0.593	1.0 (0.7; 1.5)	0.864
Length of stay >4 days								
No	2 647 (99%)	2 103 (99%)	108 (100%)	436 (99%)	1 (ref.)		1 (ref.)	
Yes	29 (1.1%)	23 (1.1%)	0 (0%)	6 (1.4%)	0.0 (0.0; ---)	0.727	1.3 (0.5; ---)	0.618
Respiratory distress								
No	2 637 (99%)	2 094 (98%)	106 (98%)	437 (99%)	1 (ref.)		1 (ref.)	
Yes	39 (1.5%)	32 (1.5%)	2 (1.9%)	5 (1.1%)	1.2 (0.3; ---)	0.774	0.7 (0.3; 1.9)	0.550
Ventilator support								
No	2 639 (99%)	2 097 (99%)	106 (98%)	436 (99%)	1 (ref.)		1 (ref.)	
Yes	37 (1.4%)	29 (1.4%)	2 (1.9%)	6 (1.4%)	1.4 (0.3; ---)	0.674	1.0 (0.4; ---)	0.991
Congenital anomaly								
No	2 669 (100%)	2 121 (100%)	108 (100%)	440 (100%)	1 (ref.)		1 (ref.)	
Yes	7 (0.3%)	5 (0.2%)	0 (0%)	2 (0.5%)	0.0 (0.0; ---)	0.879	1.9 (0.4; 10)	0.434

[1]OR: Odds ratio; CI: confidence interval.;
